# A Human Microbiota-Associated Murine Model for Assessing the Impact of the Vaginal Microbiota on Pregnancy Outcomes

**DOI:** 10.3389/fcimb.2020.570025

**Published:** 2020-10-06

**Authors:** Alexandra A. Wolfarth, Taylor M. Smith, David VanInsberghe, Anne Lang Dunlop, Andrew S. Neish, Elizabeth J. Corwin, Rheinallt M. Jones

**Affiliations:** ^1^Department of Pathology, Emory University School of Medicine, Atlanta, GA, United States; ^2^Emory University Nell Hodgson Woodruff School of Nursing, Emory University School of Medicine, Atlanta, GA, United States; ^3^Division of Pediatric Gastroenterology, Hepatology, and Nutrition, Department of Pediatrics, Emory University School of Medicine, Atlanta, GA, United States

**Keywords:** vaginal microbiota, bacterial vaginosis (BV), humanization, pregnancy, inflammation

## Abstract

Disease states are often linked to large scale changes in microbial community structure that obscure the contributions of individual microbes to disease. Establishing a mechanistic understanding of how microbial community structure contribute to certain diseases, however, remains elusive thereby limiting our ability to develop successful microbiome-based therapeutics. Human microbiota-associated (HMA) mice have emerged as a powerful approach for directly testing the influence of microbial communities on host health and disease, with the transfer of disease phenotypes from humans to germ-free recipient mice widely reported. We developed a HMA mouse model of the human vaginal microbiota to interrogate the effects of Bacterial Vaginosis (BV) on pregnancy outcomes. We collected vaginal swabs from 19 pregnant African American women with and without BV (diagnosed per Nugent score) to colonize female germ-free mice and measure its impact on birth outcomes. There was considerable variability in the microbes that colonized each mouse, with no association to the BV status of the microbiota donor. Although some of the women in the study had adverse birth outcomes, the vaginal microbiota was not predictive of adverse birth outcomes in mice. However, elevated levels of pro-inflammatory cytokines in the uterus of HMA mice were detected during pregnancy. Together, these data outline the potential uses and limitations of HMA mice to elucidate the influence of the vaginal microbiota on health and disease.

## Introduction

The vagina houses a numerically immense and functionally consequential microbiota (Mendling, 2016; Kaminska and Gajecka, 2017). Distinct anatomic regions within the female reproductive tract house a dynamically changing microbial community of vastly different numbers and taxonomic composition. For example, it is well-known that the vagina maintains a numerically vast microbiota while the uterus (pregnant and non-pregnant) is normally colonized with very limited microbiota (Aagaard et al., 2014; Franasiak and Scott, 2017). Lactobacilli are the dominant taxa of the human vaginal microbiota and are one of the first bacteria to which neonates are exposed. The origin and consequences of the unique lactobacilli-dominant community structure remains enigmatic, though it is generally accepted that lactate produced by these bacteria results in the characteristic acidic pH of the healthy vagina. This is a result of direct or syntrophic fermentation of the abundant glycogen found in apical squamous epithelia of the vagina (Charbonneau et al., 2016; Reid, 2016). This low pH is generally assumed to have intrinsic bacteriostatic effects and is a prime example of the innate defenses of the female reproductive tract. Thus, the female reproductive tract has a highly adapted microbiota with known beneficial effects including colonization resistance against pathogens (Sykiotis and Bohmann, 2008).

In support of this notion, a study that characterized the diversity of bacterial taxa within the vaginal microbiome of nearly 400 multi-ethnic reproductive age women discovered that the vaginal microbiota clustered into community state types (CSTs) (Ravel et al., 2011; Gajer et al., 2012). Indeed, about 25% of the women sampled clustered into a group (designated CST IV) where lactobacilli was not the dominant taxon. This group was associated with a less acidic pH and higher indices associated with bacterial vaginosis (BV). Intriguingly, African American and Hispanic women were overrepresented among CST IV. Additionally, African Americans are also at a higher risk for adverse pregnancy outcomes, including preterm birth (Kessel et al., 1988). Considering the association between BV and preterm birth (Goldenberg et al., 1998; Leitich et al., 2003), investigating the microbiota of the reproductive tract in this context may offer a path toward refined associations and molecular mechanisms underlying the development of adverse pregnancy outcomes in this population of women.

BV is a common clinical syndrome seen in gynecological practice. African American women are more commonly affected by BV, with prevalence estimates of 51.4% for African American women compared to 23.2% for US white women of reproductive age (Koumans et al., 2007). This condition is best conceptualized as an ecological disorder characterized by the displacement of a lactobacillus-dominant microbiota (and loss of acidic pH) by a variable mix of facultative organisms often including *Gardnerella vaginalis* (Nasioudis et al., 2017). Interestingly, BV can result in the formation of endometrial biofilms with *G. vaginalis*, observed in 50% of patients with BV, including both pregnant and non-pregnant patients (Swidsinski et al., 2013), indicating abnormal microbial community structure can result in ascending infection. Furthermore, a consequence of reduced lactobacilli abundance in the vaginal canal is associated with reduced implantation efficiency in humans (Moreno et al., 2016). These observations suggest that specific commensal taxa such as lactobacilli within the female reproductive tract mediate colonization resistance against pathogens and positively influence pregnancy outcomes.

Despite the known complications in patients with BV, animal models for human BV remain underdeveloped. To address this void, we aimed to develop a human microbiota-associated (HMA) mouse model of BV using germ-free mice. While the natural microbiota of mice differs greatly to that of humans (Ley et al., 2005), HMA mice allows microbial colonization of germ-free mice with the relevant human microbiota and later studied. Colonizing germ-free mice with bacteria from human donors aims to maintain a microbiota diversity and profile similar to the human donor and thus may be a faithful model to examine the role of the human vaginal microbiota in diseases of the reproductive tract and adverse pregnancy outcomes (Marcobal et al., 2013). Furthermore, housing of HMA mice in a hermetically sealed bio-exclusion cage system means that no further bacteria from the environment will enter and alter the diversity of the humanized mouse (Paik et al., 2015).

Herein, we describe the generation of HMA mice harboring the microbiota collected from the vaginal tract of pregnant women and describe the extent to which the human vaginal microbiota can colonize the vaginal tract of germ-free mice. We show that pregnant women with BV harbor a distinct microbiota and variable risk for adverse pregnancy outcomes. Further, we observed substantial variation in pregnancy outcomes and pro-inflammatory cytokine production among HMA mice, despite donor microbiota being a poor predictor of these properties. Together, these data outline the potential use and limitations of using HMA mice harboring the microbiota collected from the human vaginal tract to elucidate the impact of the vaginal microbiota on pregnancy outcomes.

## Results

### Pregnant Women With BV Harbor a Distinct Microbiota Community Structure

**Figure 1:**
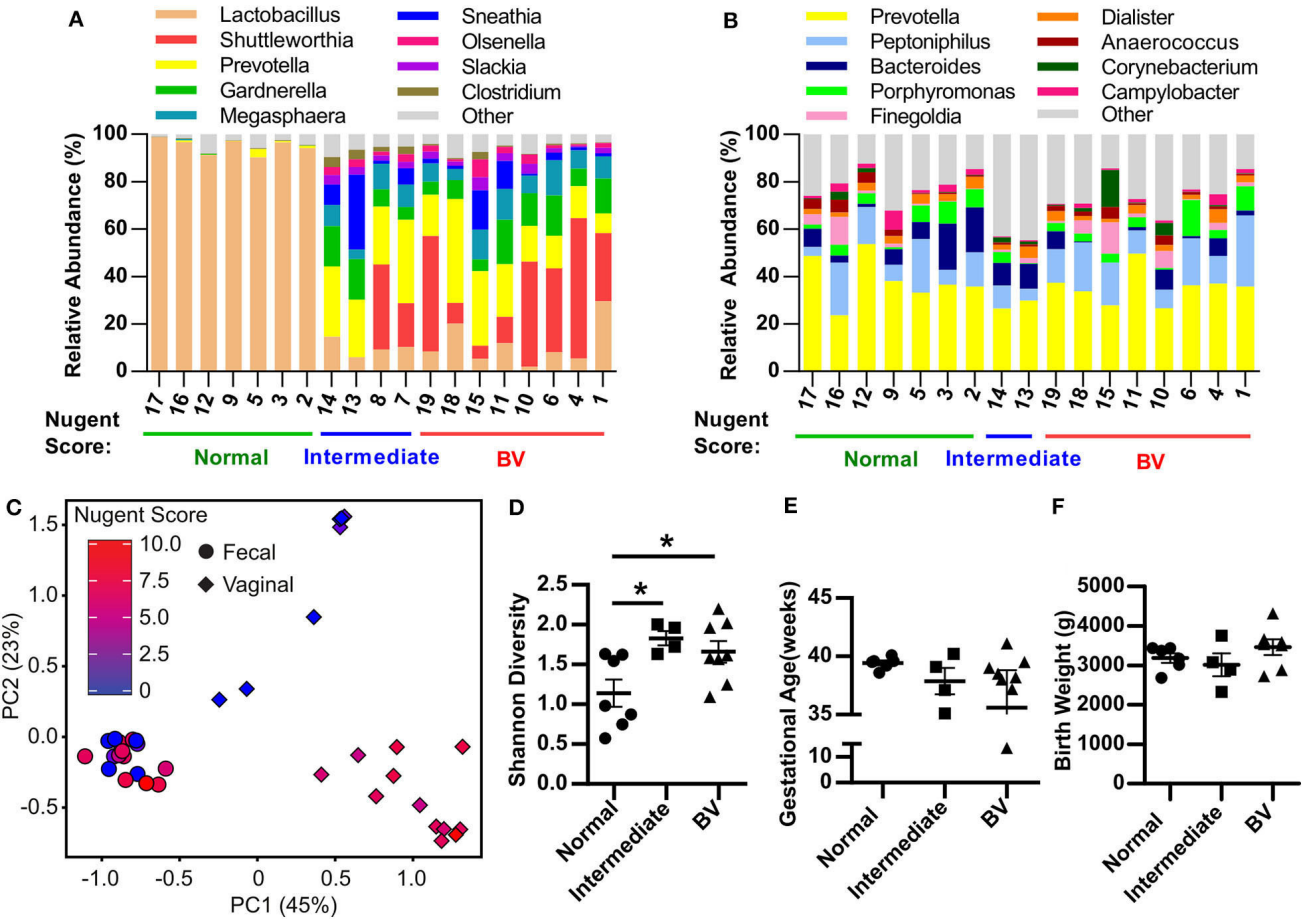
Pregnant women with bacterial vaginosis harbor a distinct microbiota community structure. **(A,B)** Relative abundance of bacterial genera within the vaginal tract **(A)** and rectum **(B)** of pregnant women assigned as Normal, Intermediate, or BV by Nugent score described in Table 1. Data represents the top 10 most abundant bacterial genera detected. Each column represents one patient. **(C)** Principal Component Analysis (PCA) plot depicting the beta-diversity of the microbiota community structure within the vaginal tract and the rectum of pregnant patients described in Table 1. **(D)** Shannon diversity index of patient vaginal microbiota determined via 16S rRNA gene sequencing according to patient Nugent score. Data graphed as mean ± SEM. **(E)** Gestational age of delivery for pregnant patients described in Table 1 with either a normal, intermediate or BV Nugent score. Data graphed as mean ± SEM. **(F)** Birth weight of infant delivered by patients described in Table 1 with a normal, intermediate or BV Nugent score. Data graphed as mean ± SEM. Statistical significance determined via One-way ANOVA, Turkey's multiple comparison test **(D,F)** or Kruskal-Wallis test, Dunn's multiple comparisons test **(E)**. *p < 0.05. *n* = 19 patients.

To employ a rigorous experimental approach to generate HMA mice, it is essential to establish a well-characterized cohort of patients with well-characterized disease states as donors of human microbiota. To this end, 19 pregnant African American women were recruited and information pertaining to Nugent score of vaginal swab were collected at the same time as the microbiota sample (for gold standard diagnosis of BV), while patient demographics, health status, pregnancy complications and birth outcomes were recorded as well (Table 1). Specifically, seven women presented with a normal Nugent score between 0 and 3, four women presented with an intermediate Nugent score between 4 and 6, and eight women presented with a Nugent score of 7 or higher indicative of BV (Table 1). Seven women experienced a urogenital infection during gestation, with five of those women assigned intermediate or BV Nugent scores at the time of swab collection (Table 2). Of the five women diagnosed with a urogenital infection, four of them were given an antibiotic and/or antifungal prior to the swab collection (Table 2). In addition, eight women had white/gray vaginal discharge reported at the time of sampling, with all of those women assigned either intermediate or BV Nugent scores (Table 2). The vaginal and rectal microbiota community structures were characterized for each of the 19 patients. Healthy patients with a low Nugent score harbored a microbiota diversity typical of that previously detected in heathy women (Nunn and Forney, 2016) where the microbial community is dominated by lactobacilli (Figure 1A). However, patients with an intermediate or high Nugent score harbored a dysbiotic vaginal microbiota community structure, typified by the diminishment in the relative abundance of lactobacilli and an expansion in the relative abundance of bacteria of genera Prevotella, Gardernella, and Shuttleworthia (Figure 1A). Furthermore, beta diversity analysis of the vaginal microbiota revealed distinct separation of patients with respect to their Nugent scores (Figure 1C). By contrast, characterization of the fecal microbiota diversity of these patients did not reveal any salient differences in either relative bacterial abundances (Figure 1B), nor in beta diversity, with no detectable separation of patients clustering with respect to their Nugent score (Figure 1C). The Shannon diversity index of the vaginal microbiota of each patient was also plotted with respect to the grouped Nugent score, and revealed that patients with a normal Nugent score between 0 and 3 had significantly lower microbiota diversity (Shannon diversity index) compared to patients with an intermediate Nugent score between 4 and 6, or compared to patients with a Nugent score of 7 or higher (Figure 1D). The gestational age and birthweight of each infant was also collected and revealed that while there was some variation among the infants, our total sample size of 19 did not demonstrate a statistically significant difference among infants carried by women with a normal, intermediate or high Nugent score (Figures 1E,F). Together, these data establish a cohort of normal and disease patients with defined and quantifiable disease activity for use in the generation of HMA mice.

**Table 1:** Clinical parameters of the 19 pregnant women used for HMA mouse generation.

**Characteristics**	**Subjects (*n* = 19)**
Age, years (mean ± sd)	25.1 ± 5.06
**Race/Ethnicity**	
African American	19 (100%)
**Educational level**	
Less than high school	6 (31.6%)
High school or GED	6 (31.6%)
Some college	6 (31.6%)
College graduate	1 (5.3%)
**Prenatal Insurance**, ***n*** **(%)**	
Medicaid	17 (89.5%)
Private	2 (10.5%)
**Nugent Score**, ***n*** **(%)**	
Normal (0–3)	7 (36.8%)
Intermediate (4–6)	4 (21.1%)
BV (7+)	8 (42.1%)
Gestational Hypertension	1(5.3%)
Gestational Diabetes	1(5.3%)
**Obstetrical history**, ***n*** **(%)**	
Prior term birth	11 (57.9%)
Prior preterm birth	3 (15.8%)
Birth Outcome^*^, ***n*** **(%)**	
Full term	10 (52.6%)
Early term	6 (31.6%)
Preterm	1(5.3%)
Spontaneous abortion	1(5.3%)
**Exposure to antibiotics during pregnancy**, ***n*** **(%)**	
Yes	7 (36.8%)
No	12 (63.2%)

* *Full term (39 weeks≥), Early term (39 weeks < x ≤ 36 weeks), Preterm (36 weeks <)*.

**Table 2:** Urogenital infection and antibiotic/antifungal use among the 19 pregnant women during pregnancy.

**Patient #**	**Nugent score**	**Vaginal discharge** **color**	**Infection during** **pregnancy**	**Antibiotic**	**Antifungal**	**GA at Antibiotic and/or** **Antifungal Use (weeks)**
1	8	White/Gray				
2	1	NR				
3	1	Clear				
4	7	White/Gray	BV	Cleocin		12.2*
5	0	Clear				
6	7	White/Gray	Trichomoniasis	Flagyl		30
7	5	Clear	Chlamydia	Zithromax		12
8	6	White/Gray	Gonorrhea	Ceftriaxone		10*
9	0	Clear	GBS UTI	Cephalexin	Clotrimazole	6.5*
10	7	White/Gray				
11	8	White/Gray				
12	0	NR	BV	Flagyl		34.5
13	6	NR				
14	5	NR				
15	7	White/Gray				
16	0	Clear				
17	0	Clear				
18	8	White/Gray	Trichomoniasis	Flagyl		8*
19	10	NR				

*NR, Not Recorded; GA, Gestational Age. Asterisk denotes antibiotic/antifungal use prior to vaginal swab collection*.

### Generation of Human Microbiota-Associated (HMA) Mice Harboring the Microbiota Collected From the Vaginal Tract of Pregnant Women With Bacterial Vaginosis

**Figure 2:**
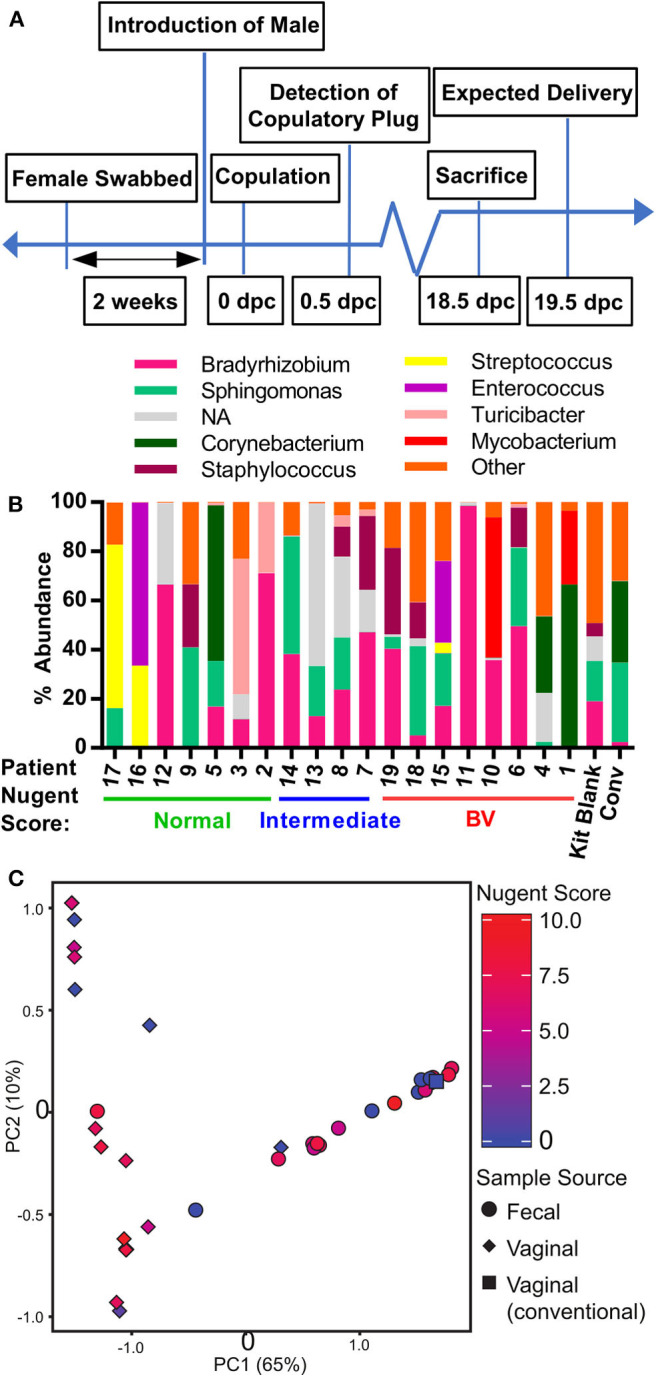
Generation of human microbiota-associated (HMA) mice harboring the microbiota collected from the vaginal tract of pregnant women with bacterial vaginosis. **(A)** Graphical depiction of experimental approach to generate human microbiota-associated (HMA) mice harboring the microbiota collected from the vaginal tract of pregnant patients described in Table 1. Swabs were collected from the vaginal tract and immediately transported to the Emory Gnotobiotic Animal Core (EGAC). The vaginal tract of female germ-free C57BL/6 were inoculated by physically wiping the swab on the vaginal opening of the mouse. Mice were then housed in Tecniplast ISOcageP Bioexclusion cages for the microbiota to colonize. After 2 weeks, a male germ-free mouse was introduced to the HMA female mouse and conception monitored. On 18.5 dpc (days post-coitum), pregnant female mice were sacrificed under sterile conditions for sample collection and analysis. **(B)** Relative abundance of the bacterial genera detected via 16S analysis in the vaginal tract of HMA mice on 18.5 dpc. Data represents the top 10 bacterial genera detected and each stacked column represents one mouse. Data is separated by BV status of the corresponding human donor. The total read count for each sample is provided at the top of each bar. NA (not assigned) refers to sequences that were unclassifiable at this taxonomic level. **(C)** Principal Component Analysis (PCA) plot depicting the beta-diversity of the microbiota community structure within the vaginal tract and gastrointestinal tract of HMA mice on 18.5 dpc. Symbols are colored by the Nugent score of the corresponding human donor.

The patients' vaginal microbiota was swabbed at the hospital and the swabs were transported immediately to the Emory Gnotobiotic Animal Core (EGAC). The vaginal tract of female germ-free C57BL/6 mice was inoculated by physically wiping the vaginal swab on the mouse, concentrating the swab to the vaginal opening. This process was typically completed within 2 h of collecting the vaginal swab from the patient. Mice were then housed within bioexclusion husbandry cages for the vaginal microbiota to establish and colonize. After 2 weeks, a male germ-free mouse was introduced into the bioexclusion cages of each HMA female mouse, and conception date recorded by the appearance of a viscous copulatory plug. At 18.5 days post-coitum (18.5 dpc) and before delivery of litters, mice were sacrificed and the uterus, vagina, and fecal pellet collected under sterile conditions (Figure 2A). Characterization of the vaginal microbiota in HMA mice using 16S rDNA amplicon sequencing revealed that although each HMA mouse became colonized by microbes, the proportional abundance of those taxa in the HMA mice was considerably different to the donor sample (Figures 1A, 2B). Importantly, the lactobacilli from the normal Nugent score patients did not efficiently colonize the mouse vaginal tract (Figure 2B). To compare the vaginal microbiota of the HMA mice to the vaginal microbiota of a pregnant conventionally raised mouse harboring an undisturbed microbiota, a female mouse housed in the adjacent specific pathogen-free murine vivarium was collected at 18.5 dpc. Microbiota analysis revealed the conventional mouse had a vaginal microbiota abundant in Sphingomonas and Corynebacterium (Figure 2B). During microbial DNA isolation and PCR amplification, a blank sample was included among our mouse vaginal samples to determine any environmental contaminants in our samples (denoted “kit blank”). While the kit blank contained some bacteria that were also found in our HMA mouse samples, the total number of reads obtained after PCR amplification was markedly less compared to those found in the HMA samples or the conventional sample (Figure 2B). Furthermore, there was high variability in the colonizing microbiota of each HMA mouse, and similarity between the microbiota of HMA mice was not driven by the BV status of the donor sample (Figure 2C). We also detected no significant differences in the fecal microbiota of the HMA mice and no separation based on patient Nugent score (Figure 2C). These results suggest that while the vaginal canal of germ-free mice provides a competition-free niche for bacteria in a donor sample, the vaginal microbiota of HMA mice generated using this method of inoculation does not closely resemble the community present in donor samples.

### Pregnancy Outcomes in Human Microbiota-Associated (HMA) Mice Harboring the Microbiota Collected From the Vaginal Tract of Pregnant Women With Bacterial Vaginosis

**Figure 3:**
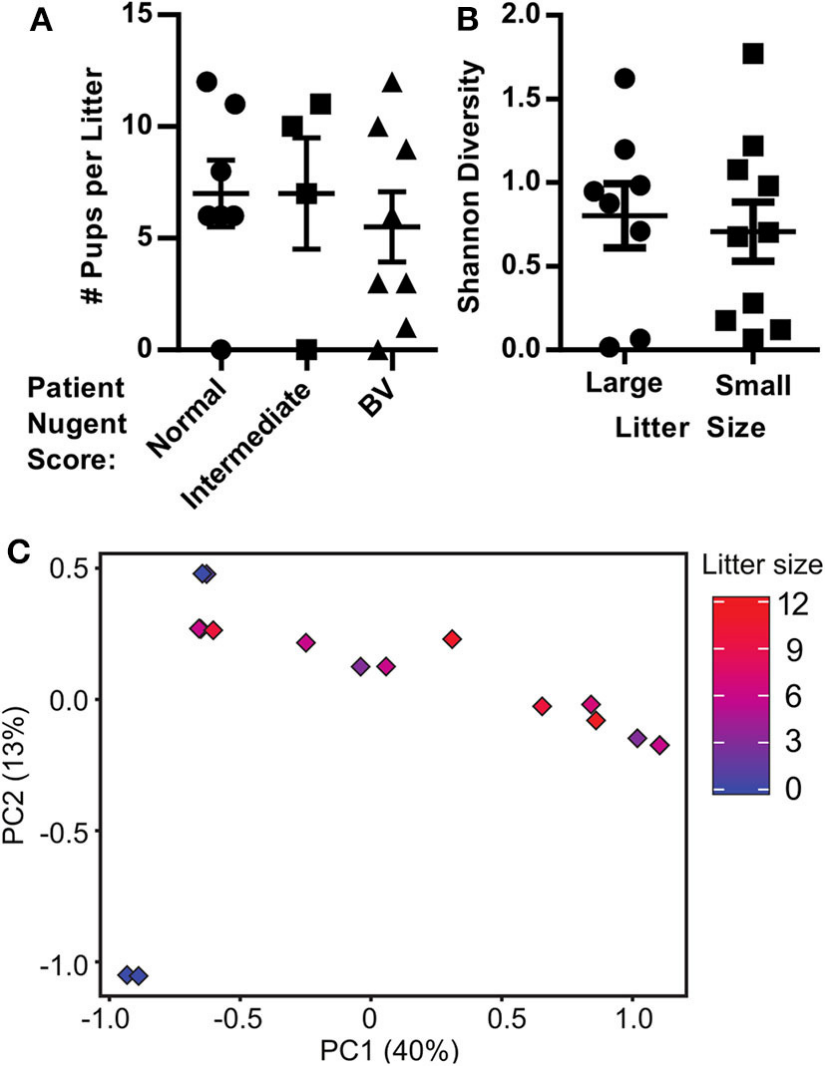
Pregnancy outcomes in human microbiota-associated (HMA) mice harboring the microbiota collected from the vaginal tract of pregnant women with bacterial vaginosis. **(A)** The number of pups detected in the uterine horns of HMA mice on 18.5 dpc separated by the Nugent score of the corresponding donor. Data graphed as mean ± SEM. **(B)** Shannon diversity index of the mouse vaginal microbiota determined via 16S analysis on 18.5 dpc separated by litter size of the pregnant HMA mouse. Less than 7 pups on 18.5 dpc is considered a small litter, while 7 or more pups is considered a large litter. Data graphed as mean ± SEM. **(C)** Principal Component Analysis (PCA) plot depicting the beta-diversity of the microbiota community structure within the vaginal tract of HMA mice on 18.5 dpc. Symbols are colored by the litter size on 18.5 dpc.

Despite no similarity between donor and HMA mouse vaginal microbiota, we recorded considerable variation in the number of pups *in utero* among the HMA mice and sought to determine whether the microbiota harbored in the vaginal canal correlated with litter size. We determined litter size in HMA mice by recording the number of pups within the uterine horns at 18.5 dpc. This was done before birth in order to obtain a faithful count of viable pups and to mitigate the prospect that the new moms' cannibalize their newborn offspring, which often occurs postpartum in C57BL/6 mice and could affect our data. We compared the litter size at 18.5 dpc with the Nugent score of the corresponding patient described in Table 1. We detected considerable variation in litter size, but no significant difference in litter size between HMA mice colonized with normal, intermediate or BV patient swabs (Figure 3A). We also compared the litter size of HMA mice with the Shannon diversity index of their vaginal microbiota and found that mice with smaller litter sizes did not have a significant difference in vaginal microbiota diversity (Figure 3B). Lastly, a PCA plot comparing the beta diversity of the mouse vaginal microbiota and the corresponding litter size (Figure 3C) shows that the vagina microbiota of the HMA mice did not have a significant influence on litter size.

### Altered Pregnancy Outcomes in Human Microbiota-Associated (HMA) Mice Is Associated With Elevated Levels of Pro-inflammatory Cytokines in the Uterus of Mice During Pregnancy

**Figure 4:**
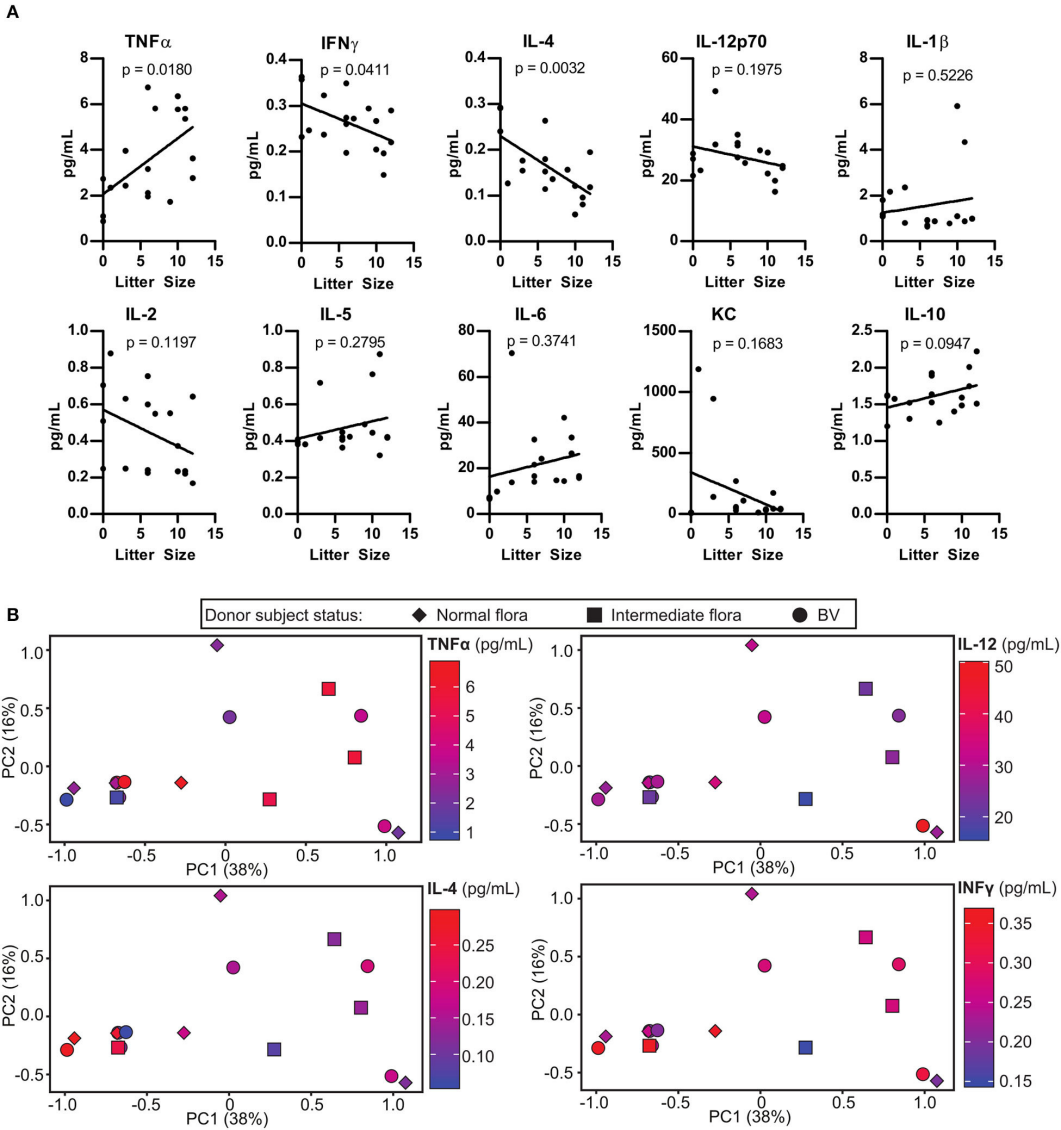
Pregnancy outcome in human microbiota-associated (HMA) mice is associated with altered uterine cytokine levels during pregnancy. **(A)** Cytokine concentrations in the uterus of HMA mice on 18.5 dpc. Cytokine levels are plotted with respect to the litter size. Statistical significance determined using linear regression analysis, *p*-value is indicated on the figure for each cytokine. **(B)** Principal Component Analysis (PCA) analysis depicting the correlation between cytokines concentrations in the uterus that were significantly altered or trending toward significance in **(A)**, the HMA mouse vaginal microbiota community, and the Nugent score of the corresponding donor patient.

Inflammation in the uterus, or endometritis is commonly associated with pregnancy complications. We examined the extent to which the colonizing microbes influenced levels of inflammation in the uterus of HMA mice. Although the uterus has considerably lower levels of bacteria compared to the vaginal tract, it may be possible that certain bacterial species in the vaginal tract have negative impacts on the physiology of the entire reproductive system. Linear regression analysis of cytokine concentrations in the uterus of HMA mice described in Figure 2 revealed a significant negative correlation between IFNγ and IL-4 with litter size, whereas a significantly positive correlation was found with TNFα levels (Figure 4A). To determine the extent to which certain murine vaginal microbial communities were associated with altered uterine cytokine levels during pregnancy, we conducted PCA analysis comparing cytokine levels and the vaginal microbiota community. However, our analysis revealed that the HMA mouse vaginal microbiota did not group with any alterations in uterine cytokine levels and had no correlation with BV status of the patient (Figure 4B).

## Discussion

In order to better understand the effect of the vaginal microbiota on health, we assessed the utility of the HMA mouse approach, which has been widely employed in the study of host cell and intestinal microbe interactions (Marcobal et al., 2013; Ridaura et al., 2013). Studies using HMA mice have not only attributed causality of specific microbial community structures in the development of many chronic diseases such as metabolic syndrome, but have also helped to unravel the mechanisms behind these associations- an approach that is unfeasible in human subjects (Chassaing et al., 2015). While mice with a humanized microbiota have offered an invaluable model for the study of the gastrointestinal microbiota and its role in human health, the same mouse model for the human vaginal microbiota has not been fully established (Bradshaw and Sobel, 2016). We first collected patient data on a cohort of pregnant women, including Nugent scores for the assessment of BV in patients. Using vaginal swabs from these patients, we generated HMA mice harboring the microbiota collected from the vaginal tract of pregnant women with BV. Comparison of the vaginal microbiota of BV patients and HMA mice revealed no immediately apparent similarity. In addition, there was no correlation detected between the number of pups within pregnant HMA mice and Nugent scores from BV patients. Significantly elevated levels of pro-inflammatory cytokines in the uterus of mice were detected during pregnancy, although there was no clear correlation between the murine vaginal microbiota, Nugent scores from BV patients and pro-inflammatory cytokine levels.

In both mice and humans, the uterus functions to nurture a fertilized egg until the fetus, or offspring, is ready to be delivered. However, the anatomy of the mouse female reproductive tract has clear differences compared to humans. The murine uterus is bicornuate, forming two uterine horns that help accommodate large litter sizes, with an average of 6.2 newborns per litter (Nagasawa et al., 1973). By comparison humans have a simplex uterus with a single cavity located between the bladder and the rectum, and typically harbors only one infant. Importantly, both humans and mice have a cervix which forms a tight physical barrier between the vagina and uterine cavity. The cervix plays a critical role in preventing vaginal bacteria from ascending into the uterus (Racicot et al., 2013; Pavlidis et al., 2020). As in humans, mice have a resident commensal vaginal microbiota and virtually undetectable numbers of microbes in the uterus (Han et al., 2004; Digiulio et al., 2010). As in humans, mice also undergo a hormonal cycle regulated by steroid hormones, with their cycle being much shorter, about 4 days in total, compared to a human's 28 day cycle (Gonzalez, 2016). Interestingly, mice also do not undergo menstruation and instead only decidualize if fertilization occurs (Finn, 1998). The lack of menstruation may be a governing determinant in the establishment of the microbiota community structure within the mouse reproductive tract. Indeed, the composition of the vaginal microbiota differ greatly between mice and humans. In fact, humans have a distinct vaginal microbiota compared to most other mammalian species sampled including even non-human primates (Miller et al., 2017). Humans are the only species to have a vaginal microbiota dominated by lactobacilli, while all other mammals have a considerably more diverse vaginal microbe community structure (Swartz et al., 2014; Yildirim et al., 2014). The teleological explanation for the specific nature of the human vaginal microbiota diversity in terms of the purpose it serves remains enigmatic, with progress in our understanding perhaps hindered by the very fact that there is no suitable animal to model the human vaginal microbiota (Miller et al., 2016). Establishing an HMA animal model would greatly enhance our understanding of how the vaginal microbiota affects the development of several human reproductive diseases, many of which cause pre-term births, and may facilitate the development of novel therapies that are desperately needed for millions of women.

When generating the HMA mice using human vaginal swabs, ensuring viability of the bacteria present on the patient swab was critical and challenging. The vaginal swab was self-collected by the patients, stored in a sterile tube and immediately transferred to the facility housing the germ-free recipient female mice. Although we inoculated the mice without delay, about an hour would transpire between patient collection and mouse inoculation. During this time, it is unclear how many and what type of bacteria had lost viability by the time the germ-free mice were inoculated, since 16S microbiota analysis of the swabs also detects non-viable bacterial DNA. Because of this limitation, it is possible that germ-free mice are capable of harboring a vaginal microbiota similar to humans, but this particular method of inoculation using a patient swab may be not the optimal method. This challenge with viability may be a contributory factor explaining why the HMA mice did not harbor a vaginal microbiota similar to the human patient swabs. However, the total sequencing read counts of the HMA vaginal samples showed little variability, as 16/19 samples had at least 1.80 × 10^5^ total reads and were comparable to the total read count of the conventional mouse vaginal sample from the SPF facility at 7.4 × 10^5^ reads. In addition, antibiotic or antifungal use by the patient did not influence the bacterial abundance or total read count. Interestingly, many of the bacterial taxa detected in the HMA mouse vaginal samples were only present at low abundances in the patient swab. While the kit blank sample contained some of the bacterial taxa detected in the HMA mice, the kit blank had a very low read count compared to HMA mouse samples. Nevertheless, these observations do raise the possibility that environmental contaminants may be present in the HMA samples, albeit at very low levels. In addition, in future approaches, we propose that a blank microbiome sample should be generated by collecting samples from the vaginal tract of germ-free mice to detect any mouse-derived contaminants in the HMA samples. Nevertheless, for future investigations, colonization of the HMA mice may be more successful if the viability of microbes on the swab is ensured. Furthermore, determining the best method to store the patient swab during transport requires consideration given that conditions related to temperature and oxygen exposure would ultimately favor the viability of some bacteria over others. Therefore, due to the challenge of confirming the viability of all bacteria present on a patient swab, enhanced approaches to sample collection and inoculation should be considered.

As an alternative approach, and because the human vaginal community state types have been well-defined (Ravel et al., 2011), it may also be possible to isolate the major bacterial species that make up a particular vaginal community state type to generate a defined flora that may then be introduced to the germ-free mice via vaginal lavage. This approach would contribute to establishing the proof of principle that human vaginal microbiota isolates can indeed colonize the murine vaginal tract. This approach would be similar to the approach successfully implemented in the generation of mice with a standardized intestinal microbiota. For example, altered Schaedler flora (ASF) is a standardized cocktail of eight bacterial species that is employed to study gut-microbe interactions in mice (Wymore Brand et al., 2015; Lyte et al., 2019). In addition to a vaginal lavage of a relevant standardized cocktail of bacteria, the mice could also receive an oral gavage of the ASF cocktail to ensure the intestinal microbiota is comparable among all female mice. This would address any confounding effects the intestinal microbiota may have on pregnancy outcomes. Using a similar method to ASF may serve as a reliable approach to modeling the human vaginal microbiota in mice.

This study aimed to generate HMA mice to determine the effect of the vaginal microbiota on pregnancy outcomes. To measure reproductive fitness in the HMA mice, we enumerated the number of pups *in utero* at 18.5 dpc. Small litter sizes in mice have been correlated with pregnancy complications such as ascending uterine infections and dysregulation of protein expression critical to uterine remodeling and regeneration (Tuffrey et al., 1992; Zavan et al., 2016; Mccallum et al., 2018). Although we saw high variability in litter sizes among the HMA mice, smaller litter sizes did not correlate with the HMA mouse vaginal microbiota. In addition to litter size, we also measured the levels of 10 pro-inflammatory cytokines in order to detect any uterine inflammation. Chorioamnionitis, also known as intra-amniotic infection (IAI) is an inflammation of the fetal membranes due to a bacterial infection (Tita and Andrews, 2010). Up to 40% of preterm births are clinically associated with intrauterine infections (Agrawal and Hirsch, 2012). Elevated levels of pro-inflammatory cytokines such as TNFα, IL-1, IL-12, IL-6, and IL-2 have been linked to chorioamnionitis (Negishi et al., 1996; Holst et al., 2007; Berry et al., 2011; Revello et al., 2016) and therefore elevated levels of these cytokines serve to identify potential pregnancy complications. Interestingly, we saw a significant positive correlation between TNFα and litter size. While pro-inflammatory cytokines are associated with inflammation during chorioamnionitis, pro-inflammatory cytokines downstream of NF-κB activation, such as TNFα, play an important role in uterine homeostasis and initiating critical events during the estrous cycle and pregnancy (Evans and Salamonsen, 2012; Sierra-Mondragon et al., 2015). We hypothesize that the small litters during pregnancy could have altered the level of TNFα expression during pregnancy. While we did see a significant negative correlation between INFγ and IL-4 with litter size, we did not detect a correlation between these cytokine levels and the HMA mouse vaginal microbiota. The number of reabsorptions could not be enumerated in this study, but may also have contributed to differences in cytokine levels. Given the variability seen in the HMA mouse vaginal microbiota, the failure to detect correlation between litter sizes, cytokine levels and the vaginal microbiota diversity is not surprising. Upon generation of a successful mouse model of the human vaginal microbiota, several additional metrics may potentially serve to compare pregnancy outcomes in mice which include, but are not limited to the presence of bacteria in the uterus during pregnancy, weight and survival of the pups after birth, time of gestation, and the number of subsequent viable litters produced by each female mouse.

The biological mechanisms by which the normal gravid uterus protects itself from ascending infection are not fully known. Recently, it has been established that the indigenous microbiota residing on host mucosal surfaces act in concert with the host to prevent pathogenic microbial colonization in a process called colonization resistance. The colonization resistance offered by the lactobacilli-rich microbiota combined with the physical barrier of the cervix is considered to be a major defense against ascending uterine infections. Furthermore, there is increasing literature describing the influence of microbial diversity within the female reproductive tract on uterine health and disease. Indeed, our approach did discover significant variation in uterine cytokine levels of HMA mice and large range of litter sizes, despite the colonizing communities bearing limited similarity to their donor sample. In that way, the approach we describe in this study could still be a valuable method for interrogating the mechanistic details through which microbes and microbial consortia influence reproductive health. Further, although we report limited similarity based on 16S rDNA amplicon sequencing, assessing microbial community composition using metagenomics and metatranscriptomics could reveal more meaningful similarities among the colonizing microbes in HMA mice. That is, it is possible that the HMA mice with more similar litter sizes and levels of inflammatory cytokines were disproportionately colonized by microbes enriched with similar metabolic capabilities. Ultimately, the HMA mouse model we describe here is a valuable step toward developing new methods that can provide mechanistic insight into how microbe-host interactions affect reproductive health.

## Methods

### Patient Recruitment and Swab Collection

Women who participated in this study were part of the Emory University African American Vaginal, Oral, and Gut Microbiome in Pregnancy Cohort Study (Corwin et al., 2017). During the recruiting period for this study (7/31/2018 through 11/15/2018), women who were recruited for the parent study were offered the option of collecting an additional vaginal and rectal swab for participation in the present study. The study protocol was carried out in accordance with the review and approval of the Emory University Institutional Review Board and the Grady Research Oversight Committee. All women participating in this study provided written informed consent in accordance with the Declaration of Helsinki.

Pregnant women were recruited to participate in this study from the prenatal care clinics of two metropolitan hospitals in Atlanta, GA, affiliated with Emory University Woodruff Health Sciences Center: Grady Memorial Hospital, a county-supported hospital that serves as a safety net for low-income patients; and Emory University Hospital Midtown, a private hospital that serves patients from a wide economic range. Inclusion criteria were that each participant is: (1) African American by self-report; (2) Between 8 and 14 weeks' gestation (verified by clinical record) and expecting a singleton pregnancy; (3) Able to comprehend written and spoken English; (4) Between 18 and 40 years of age; (5) Experiencing no chronic medical condition or taking prescribed chronic medications (verified by prenatal record). For enrolled women, data collection consisted of completing a sociodemographic questionnaire as well as self-collecting vaginal and rectal swabs during the study visit and giving permission to complete a medical record abstraction at the end of the pregnancy. For the swab collection, participants were provided verbal and pictorial instructions directing them to obtain (in a private exam room) self-collected vaginal swabs (one for Gram staining according to Nugent's score, one for DNA extraction and 16S rRNA gene sequencing, and one for inoculation of the mouse model) and one rectal swab (for DNA extraction and 16S rRNA gene sequencing). The swabs for microbiota sequencing were Sterile Catch-All™ Sample Collection Swabs (Epicentre Biotechnologies, Madison WI) which were immediately plunged into MoBio bead tubes (MoBio Laboratories, Inc., Carlsbad, CA) and frozen upright on dry ice until transported to the lab, to be stored at −80°C until DNA extraction and preparation for vaginal microbiota measurement occurs. The swabs for vaginal Gram staining were dacron swabs that were stored in a sterile tube until transport to the Emory Clinical Microbiology Laboratory for Gram staining for Nugent criteria scoring for evaluation of BV (Nugent et al., 1991). Well-designed studies support that vaginal self-collection swabs sample the same microbial diversity as physician-collected swabs of the mid-vagina and have high overall morphotype-specific validity compared with provider-collected swabs based on microbiome analysis (Forney et al., 2010).

Maternal Medical Chart Abstraction was completed by the research team using a standardized chart abstraction tool to ascertain for the following: *Gestational age at birth*. All participants receive early pregnancy dating by last menstrual period (LMP) and/or early ultrasound, given enrollment criteria. Gestational age at birth is determined from the delivery record, based upon the date of delivery in relation to the estimated date of confinement established during the 8–14 week prenatal visit. *Complications/Type and Mode of Delivery*. Gestational diabetes, preeclampsia/eclampsia, etc., type and mode of delivery are ascertained from record review after delivery and defined according to standard clinical definitions of the American College of Obstetricians and Gynecologists. *Medication use*, including any antibiotic use, in the month prior to sampling was also ascertained.

### Generation of Human Microbiota-Associated Mice

After collection of the human vaginal swabs by hospital staff the swabs were immediately transferred to research staff who inoculated 10 week-old germ-free female single-housed in a bioexclusion microisolator cage at the Emory Gnotobiotic Animal Core (EGAC). Germ-free status of mice was confirmed by bacterial 16S rDNA PCR assay paired with anaerobic culture testing, undertaken by IDEXX BioAnalytics (Columbia, MO). To handle the mice contained in the bioexclusion cages, the cage was saturated in disinfectant and placed in a sterile biosafety hood. Gloves and forceps for mouse handling were sterilized in disinfectant before opening the cage. Inoculation was achieved by physical wiping of the vaginal swab at the vaginal opening of the mouse for several seconds, ensuring adequate transfer of microbes from the swab onto the vaginal opening of the mouse. Inoculated females were housed for 2 weeks before introduction of a germ-free male. Evidence of copulation was monitored by inspection for a vaginal copulatory plug every morning after inoculation. Observance of a vaginal plug was designated as 0.5 days post-coitum (dpc). After identification of a vaginal plug the male mouse was removed to prevent the possibility of multiple copulations. The day before expected delivery (18.5 dpc) the female mouse was sacrificed and the uterine horns, vagina and fecal pellet were collected under strict sterile conditions. The number of developing pups in the uterus were enumerated and removed. The vaginal canal was immediately processed for microbial DNA isolation and the uterine horn was flash frozen for future cytokine analysis.

### Microbial DNA Isolation and 16S Analysis

After sterile collection the vaginal canal from the HMA mouse was placed in a MagnaLyser tube (Sheikh et al., 1995) with 1 mL sterile PBS. The tube was vigorously vortexed three times for 10 seconds each to remove the mucosa and bacteria from the vaginal tissue. The 1 mL of PBS containing the vaginal mucosa and bacteria was collected and the microbial DNA was isolated using the QIAamp DNA microbiome kit (Qiagen, Hilden, Germany). Adequate and faithful amplification of the 16S rRNA V4 region required an initial PCR amplification of the near full length 16S rRNA genes using the 27F (5′- AGAGTTTGATCMTGGCTCAG−3′) and 1492R (5′- TACGGYTACCTTGTTACGACTT-3′) primer pair for 25 cycles. The resulting amplicon was then used as template for amplification of the V4 region using the 515F/806R primer pair (GTGCCAGCMGCCGCGGTAA and GGACTACHVGGGTWTCTAAT) for 30 cycles in triplicate. Each 806R primer had a unique 12 base golay barcode. The PCR products were quantified using a Qubit fluorimeter (Invitrogen, Carlsbad, CA) and ran on a bioanalyzer to confirm the presence of a single band at 350 bp and no band for the “kit-ome” control. DNA samples were pooled and sequenced using the Illumina MiSeq at the Emory Integrated Genomics Core. Sequencing data was processed using the analysis pipeline developed by Vaninsberghe et al. (2020). Briefly, primers were trimmed, allowing up to one mismatch and discarding all sequences without primers, and Dada2 was used to infer the Amplicon Sequence Variants (ASVs) (Callahan et al., 2016). Sequencing data is deposited at BioProject ID PRJNA655465 and can be retrieved at http://www.ncbi.nlm.nih.gov/bioproject/655465.

### Measurement of Uterine Cytokine Levels

The uterus from HMA mice at 18.5 dpc was collected and the pups were carefully removed. The uterine wall was flash frozen with liquid nitrogen. To ensure adequate representation of the uterine tissue in subsequent cytokine analysis, the uterine tissue was ground while frozen with mortar and pestle. Fifty milligram of tissue was then added to homogenization buffer in a Magnalyser tube and homogenized twice for 30 s at 6,500 rpm using a Magnalyser (Roche, Basel, Switzerland). Protein concentrations were quantified using the Pierce BCA assay kit and the protein concentrations were diluted and normalized for cytokine quantification using the V-PLEX Proinflammatory Panel 1 Mouse Kit (Meso Scale Discovery, Rockville, Maryland) following manufacturer's protocol with the help of the Emory Multiplexed Immunoassay Core (Emory University, Atlanta, GA).

## Data Availability Statement

The original contributions presented in the study are publicly available. This data can be found in the NCBI short read archive (SRA) under the accession number PRJNA655465.

## Ethics Statement

The studies involving human participants were reviewed and approved by Emory University Institutional Review Board and the Grady Research Oversight Committee. The patients/participants provided their written informed consent to participate in this study. The animal study was reviewed and approved by Emory University IACUC committee.

## Author Contributions

RJ and AD conceived and oversaw the project. AD and EC oversaw patient sample collections. AW and TS performed the experiments. DV, AW, and TS analyzed the data and produced the figures. RJ, AW, AD, and AN wrote the manuscript. All authors contributed to the article and approved the submitted version.

## Conflict of Interest

The authors declare that the research was conducted in the absence of any commercial or financial relationships that could be construed as a potential conflict of interest.
